# Structural Differences Influence Biological Properties of Glucosylceramides from Clinical and Environmental Isolates of *Scedosporium aurantiacum* and *Pseudallescheria minutispora*

**DOI:** 10.3390/jof5030062

**Published:** 2019-07-15

**Authors:** Adriana Caneppa, Jardel Vieira de Meirelles, Rodrigo Rollin-Pinheiro, Mariana Ingrid Dutra da Silva Xisto, Livia Cristina Liporagi-Lopes, Lauro de Souza, Maria Teresa Villela Romanos, Eliana Barreto-Bergter

**Affiliations:** 1 Departamento de Microbiologia Geral, Instituto de Microbiologia Professor Paulo de Góes, Universidade Federal do Rio de Janeiro, Rio de Janeiro/RJ 21941-901, Brazil; 2 Departamento de Análises Clínicas e Toxicológicas, Faculdade de Farmácia, Universidade Federal do Rio de Janeiro, Rio de Janeiro/RJ 21941-901, Brazil; 3 Departamento de Bioquímica e Biologia Molecular, Universidade Federal do Paraná, Curitiba/PR 81531-980, Brazil; 4 Departamento de Virologia, Instituto de Microbiologia Professor Paulo de Góes, Universidade Federal do Rio de Janeiro, Rio de Janeiro/RJ 21941-901, Brazil

**Keywords:** *Scedosporium*, Glucosylceramide, Host-Pathogen Interaction

## Abstract

*Scedosporium/Lomentospora* complex is composed of filamentous fungi, including some clinically relevant species, such as *Pseudallescheria boydii*, *Scedosporium aurantiacum,* and *Scedosporium apiospermum*. Glucosylceramide (GlcCer), a conserved neutral glycosphingolipid, has been described as an important cell surface molecule playing a role in fungal morphological transition and pathogenesis. The present work aimed at the evaluation of GlcCer structures in *S. aurantiacum* and *Pseudallescheria minutispora*, a clinical and an environmental isolate, respectively, in order to determine their participation in fungal growth and host-pathogen interactions. Structural analysis by positive ion-mode ESI-MS (electrospray ionization mass spectrometer) revealed the presence of different ceramide moieties in GlcCer in these species. Monoclonal antibodies against *Aspergillus fumigatus* GlcCer could recognize *S. aurantiacum* and *P. minutispora* conidia, suggesting a conserved epitope in fungal GlcCer. In addition, these antibodies reduced fungal viability, enhanced conidia phagocytosis by macrophages, and decreased fungal survival inside phagocytic cells. Purified GlcCer from both species led to macrophage activation, increasing cell viability as well as nitric oxide and superoxide production in different proportions between the two species. These results evidenced some important properties of GlcCer from species of the *Scedosporium/Lomentospora* complex, as well as the effects of monoclonal anti-GlcCer antibodies on fungal cells and host-pathogen interaction. The differences between the two species regarding the observed biological properties suggest that variation in GlcCer structures and strain origin could interfere in the role of GlcCer in host-pathogen interaction.

## 1. Introduction

The *Scedosporium/Lomentospora* complex consists of filamentous fungi with a worldwide distribution and association with areas impacted by humans, such as sewage and polluted soils [1,2,3]. Some species are clinically relevant, with *Pseudallescheria boydii, Scedosporium aurantiacum,* and *Scedosporium apiospermum*, being the most prevalent. *Scedosporium/Lomentospora* infections found in patients [4,5] consist of a broad spectrum of pathologies, ranging from superficial to invasive as well as disseminated infections [1]. On the other hand, some species are typically environmental, such as *Pseudallescheria minutispora*, *Pseudallescheria angusta,* and *Pseudallescheria ellipsoidea*, which are rarely associated with human infections and mostly found in soil and water [4,5].

Glucosylceramides (GlcCer) are neutral glycosphingolipids composed of a fatty acid chain and a sugar unit bound to a sphingoid base [6,7]. During the last two decades, GlcCer has been extensively described in a variety of fungi, such as *Collectotrichum gloeosporioides*, *Fonsecaea pedrosoi*, *Cryptococcus neoformans*, *Candida albicans,* and different species from the *Scedosporium/Lomentospora* complex [8,9,10,11,12,13,14]. These molecules have also been described in different *Aspergillus* species, such as *Aspergillus fumigatus*, *Aspergillus oryzae*, *Aspergillus sojae*, *Aspergillus luchuensis,* and *Aspergillus awamori*, presenting not only glucosylceramide but also galactosylceramides, showing that chemical composition may vary among fungi [15,16,17]. GlcCer has been shown to be a highly conserved structure among these fungi and to exert crucial roles in fungal morphological transitions, growth, and pathogenesis [8,9,10,11,12,13,14]. Moreover, GlcCer has been described to activate the host immune system during *C. neoformans* infection [18].

GlcCer from *Scedosporium/Lomentospora* complex has been extensively studied, and it has been already identified in different *P. boydii* strains, *S. apiospermum*, *P. angusta*, *P. ellipsoidea,* and *Lomentospora prolificans* [12,13,14,19]. The major structures found are composed of a glucose unit and fatty acid chain varying in length (C-16 or C-18) and degree of unsaturation. GlcCer is an important molecule for the germination process of *P. boydii* and *S. apiospermum* and for host-pathogen interaction and recognition by the host immune system [12,13]. The use of anti-GlcCer monoclonal antibodies (Mab) has also been described as a useful tool to localize GlcCer on the fungal cell surface and to enhance phagocytosis and killing by macrophages, presenting a protective effect for mammalian hosts [12,13].

Despite extensive studies on GlcCer over the last decades, it has never been evaluated whether the structural variation found in this molecule could influence its biological properties. In this context, this work characterized, for the first time, GlcCer from a clinical (*Scedosporium aurantiacum*) and an environmental (*Pseudallescheria minutispora*) isolate, and showed the roles of these glycosphingolipids in fungal growth, virulence, and host-pathogen interaction.

## 2. Materials and Methods

### 2.1. Microorganisms and Growth Conditions

*Scedosporium aurantiacum* IHEM 21147, a clinical strain isolated from an ulcer at the ankle region, and *Pseudallescheria minutispora* IHEM 21148, an environmental strain isolated from river sediment were used in this work. Cells were kept on Sabouraud (SAB; 2% glucose, 1% peptone, 0.5% yeast extract) agar slants as a stock culture. Mycelia were obtained by growing cells in SAB liquid culture medium for seven days at room temperature with shaking. Conidia were obtained by growing cells at 30 °C on SAB agar medium for seven days. Then, the plate surface was rinsed with phosphate-buffered saline (pH 7.2) (PBS; 10 mM NaH_2_PO_4_, 10 mM Na_2_HPO_4_, 150 mM NaCl), and the suspension was filtered through a cell strainer to remove hyphal fragments and debris. The conidia were washed three times in PBS (pH 7.2) and counted in a Neubauer chamber.

### 2.2. Mice and Peritoneal Macrophage Obtention

Balb/C mice came from the Universidade Federal do Rio de Janeiro Breeding Unit (Rio de Janeiro, Brazil). They were kept at 25 °C with free access to food and water in a 12 h light/dark cycle. The study was approved by the Institutional Committee for Animal Care and Experimentation of the Federal University of Rio de Janeiro, Rio de Janeiro, Brazil, Process Number 01200.001568/2013-87 (Comissão de Ética no Uso de Animais (CEUA) em Experimentação Científica do Centro de Ciências da Saúde da Universidade Federal do Rio de Janeiro registered at Conselho Nacional de Controle de Experimentação Animal (CONCEA)).

Peritoneal macrophages from male BALB/c mice (4–8 weeks old) were cultured in RPMI 1640 medium supplemented with 10% bovine fetal serum. Cells were counted in a Neubauer chamber, and trypan blue vital dye exclusion was used to check viability.

### 2.3. Extraction and Purification of GlcCer from S. aurantiacum and P. minutispora

*S. aurantiacum* and *P. minutispora* mycelia were cultivated at room temperature, and total lipids were extracted using chloroform: methanol at 2:1 and 1:2 (*v*/*v*) ratios. The crude lipid extract was partitioned according to Folch and colleagues [20]. Lipids from the Folch lower layer were submitted to a silica gel column, which was sequentially eluted with chloroform, acetone, and methanol. Glycosphingolipids were detected in the acetone and methanol fractions, which were then purified by silica gel column chromatography, as described by Calixto and colleagues [14].

### 2.4. ESI-MS Analysis of GlcCer from S. aurantiacum and P. minutispora

MS analysis was performed in a Quattro-LC electrospray ionization mass spectrometer (ESI-MS) (Waters, Milford, MA, USA) with a triple-quadrupole mass analyzer operating at atmospheric pressure ionization (API) and assisted by a syringe pump (KD Scientific, Holliston, MA, USA) for sample infusion, according to Xisto and colleagues [19].

### 2.5. Generation of Mab Anti-GlcCer from A. fumigatus

Monoclonal antibodies against GlcCer were produced by immunizing six-week-old female BALB/c mice with purified GlcCer from *A. fumigatus*, as previously described [10].

### 2.6. Immunofluorescence Analysis

*S. aurantiacum* and *P. minutispora* cells, fixed in 4% paraformaldehyde cacodylate buffer (0.1 M, pH 7.2) for 1 h at room temperature, were blocked using PBS-1%BSA for 1 h at 37 °C. Then, either anti-GlcCer Mab or an isotype-matched control (50 mg/mL in PBS-1%BSA) was used to check GlcCer exposure on the fungal surface. After washing, cells were treated with Alexa Fluor 546-conjugated donkey anti-mouse IgG (h4l) (Invitrogen Molecular Probes, Carlsbad, CA, USA) at 1:400 dilution in PBS-1% BSA for 1 h at 37 °C. Cells were washed three times and suspended in 0.01 M *N*-propyl gallate diluted in PBS: glycerol (1:1, *v*/*v*). The suspension was applied to a microscope slide, and cells were visualized using an Olympus AX70 fluorescence microscope (Olympus America Inc., Center Valley, PA, USA) using a 620 nm filter and a 100× magnification lens.

### 2.7. Germination Assay

The germination assay of *S. aurantiacum* and *P. minutispora* conidia in the presence of anti-GlcCer Mab or an unrelated IgG as control was performed according to Rollin-Pinheiro and colleagues [13]. To check cell viability, 2,3-Bis(2-methoxy-4-nitro-5-sulfophenyl)-2H-tetrazolium-5-carboxanilide inner salt (XTT, from Sigma- Aldrich Co., St. Louis, MO, USA) was added to each well, and the plates were incubated at 37 °C for 3 h. Then, the absorbance was measured at 570 nm in a spectrophotometer.

### 2.8. Activation of Peritoneal Macrophages

#### 2.8.1. Neutral-Red Uptake Assay

Peritoneal macrophages were treated with *S. aurantiacum* or *P. minutispora* GlcCer (200, 100, 50, 25, 12.5, 6.2, and 3.1 µg/mL). After a 48-h treatment, cell viability was measured by neutral-red dye uptake [21].

#### 2.8.2. Phagocytosis Assay

Phagocytosis of *S. aurantiacum* and *P. minutispora* conidia by peritoneal macrophages obtained from BALB/c mice, after conidia were incubated with anti-GlcCer Mab or unrelated IgG control, was determined according to Xisto and colleagues [22]. The phagocytosis index was calculated as a ratio of the number of intracellular conidia to the number of macrophages counted.

#### 2.8.3. Killing Assay

Intracellular survival of *S. aurantiacum* and *P. minutispora* conidia in peritoneal macrophages, after incubation of the conidia with anti-GlcCer Mab unrelated IgG, was evaluated according to Xisto and colleagues [22].

#### 2.8.4. Nitric Oxide and Superoxide Production

Conidia and purified GlcCer were added in 96-well polystyrene tissue-culture plates containing 10^5^ peritoneal macrophages per well. After incubation for 2 h at 37 °C in the presence of 5% CO_2_, aliquots from the supernatant were collected. Nitric oxide levels were measured using the Griess reagent kit (Promega, Madison, WI, USA), and superoxide dismutase activity was determined using an MTT reduction assay in which MTT is reduced to formazan by superoxide generated from macrophages. The reduction was measured in a spectrophotometer at 570 nm. Lipopolysaccharide (LPS) and LPS inactivated by polymyxin (LPS + poly) were used as controls.

### 2.9. Statistical Analysis

Statistical analyses were done using GraphPad Prism version 5.00 for Windows (GraphPad Software, San Diego, CA, USA). One-way analysis of variance using a Kruskal-Wallis nonparametric test was used to compare the differences between groups, and individual comparisons of groups were performed using a Bonferroni posttest. The t-test was used to compare the number of colony forming units (CFU) for different groups. The 90–95% confidence interval was determined in all experiments.

## 3. Results

### 3.1. Structural Analysis of P. minutispora and S. aurantiacum GlcCer

**Figure 1:**
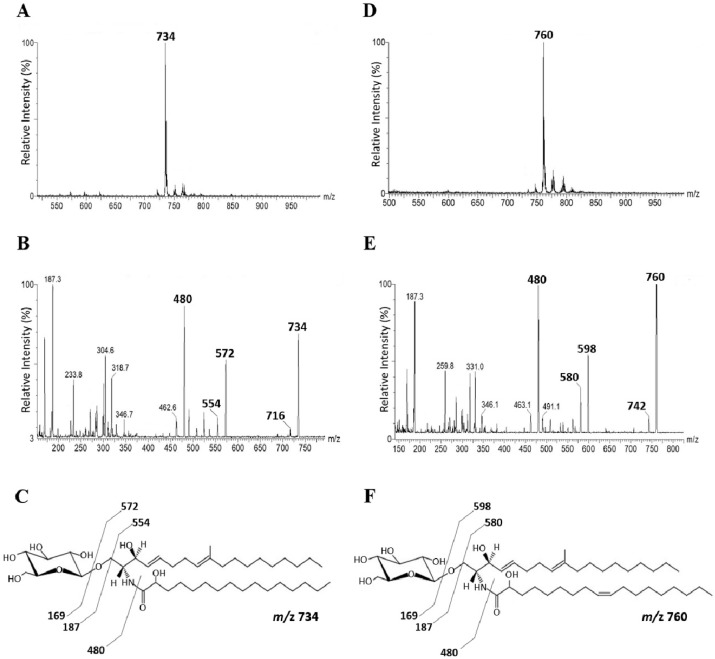
Positive ESI-MS (electrospray ionization mass spectrometer) [M+Li] analysis of GlcCer (Glucosylceramide) species from *P. minutispora* and *S. aurantiacum*. ESI-MS1 of GlcCer species from *P. minutispora* (**A**) and *S. aurantiacum* (**B**). ESI-MS2 of the major ion species *m/z* 760 (**C**) and *m/z* 734 (**D**). Proposed structures for *P. minutispora* GlcCer species (**E**) and *S. aurantiacum* GlcCer species (**F**).

GlcCer, purified from both *P. minutispora* and *S. aurantiacum,* is composed of a major molecular species at mass to charge ratio (*m/z*) 760 [M+Li]+ and *m/z* 734 [M+Li]+, respectively. (Figure 1A,B). When subjected to ESI-MS/MS, these species gave rise to major daughter ions at *m/z* 598 and 572, corresponding to the loss of hexose (162 mass units) [M- hexose- H_2_O +Li]. These daughter ions were assigned to the ceramide moieties of the GlcCers species (Figure 1C,D). The ceramide ion species at *m/z* 598 and *m/z* 572 could be assigned as *N*-2′- hydroxyoctadecenoyl- 9-methyl- 4,8-sphingadienine and *N*-2′- hydroxyhexadecanoyl - 9-methyl- 4,8-sphingadienine, respectively (Figure 1E,F). Glucose was the only monosaccharide identified by high performance thin layer chromatography (HPTLC) after hydrolysis of the glycosphingolipids with 3 M trifluoracetic acid (TFA) (data not shown).

### 3.2. Binding of Mab to Fungal Cells

**Figure 2:**
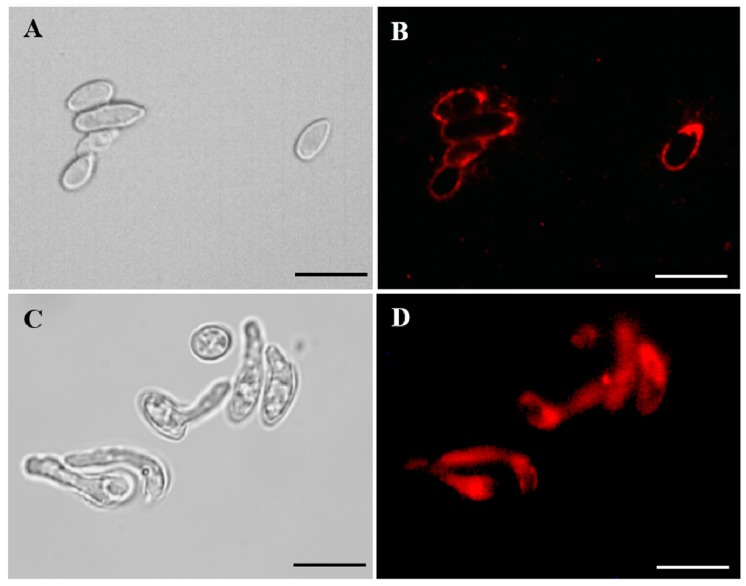
Indirect immunofluorescence of *P. minutispora* (**A**,**B**) and *S.aurantiacum* (**C**,**D**) conidial forms with anti-GlcCer Mab (monoclonal antibody). Differential interferential contrast microscopy (DIC) (**A** and **C**) and immunofluorescence (**B** and **D**). Scale bars: 10µm.

Binding of Mab to *S. aurantiacum* and *P. minutispora* cells was analyzed using an indirect immunofluorescence assay. Conidia from both species were recognized by the anti-GlcCer Mab (Figure 2), indicating that GlcCer is a structure conserved between both species. Interestingly, *S. aurantiacum* germ tubes, but not those from *P. minutispora*, were also recognized by the Mab, suggesting that GlcCers are differently exposed on the surface of *S. aurantiacum* and *P. minutispora* (Figure 2C,D).

### 3.3. Influence of Anti-GlcCer Mab on Fungal Differentiation and Viability

**Figure 3:**
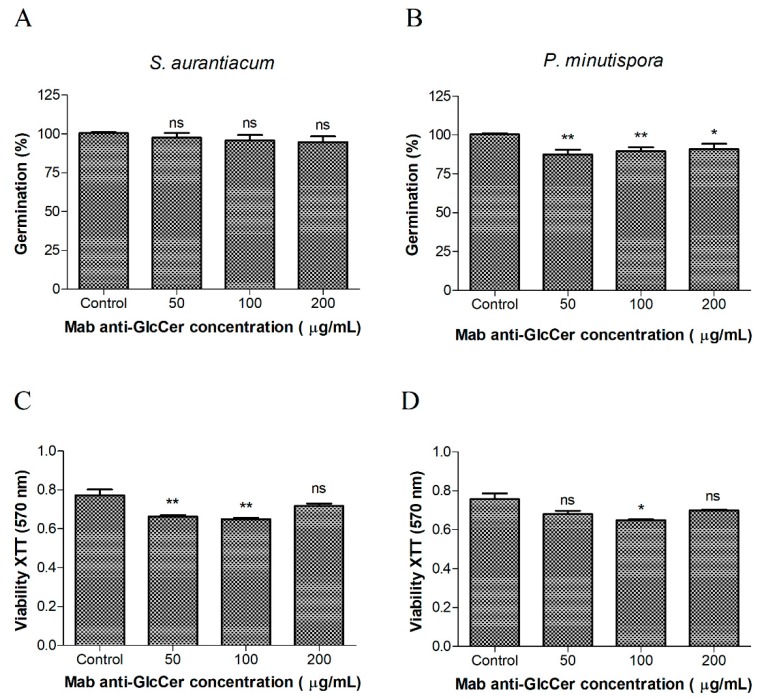
Effect of the anti-GlcCerMab (monoclonal antibody) on the germination and viability of conidial cells. Germinated conidia of *S. aurantiacum* (**A**) and *P. minutispora* (**B**) in the presence of different concentrations of anti-GlcCer Mab over 24 h were counted by optical microscopy. At least 100 conidia per field were counted, and the mean value of three independent counts was calculated. Viability assays were performed using *S. aurantiacum* (**C**) and *P. minutispora* (**D**) conidia and the anti-GlcCer Mab over 24 h and evaluated using XTT-reduction assay. The absorbance at 570 nm was measured using a spectrophotometer. * *p* < 0.05, ** *p* < 0.03.

A germination assay was performed to check whether Mab interferes in fungal morphological transition. Conidia were incubated with anti-GlcCer Mab (50, 100, and 200 µg/mL) for 4 h (*S. aurantiacum*) and 10 h (*P. minutispora*) since their germination time is different. Mab did not influence the germination process of *S. aurantiacum* and *P. minutispora* at any concentration tested (Figure 3A) since the conidia-mycelium transition was similar to the control level. On the other hand, the viability evaluation through MTT-reduction assay revealed decreasing cell viability at Mab concentrations of 50 and 100 µg/mL, for *S. aurantiacum*, and only at 100 µg/mL for *P. minutispora* (Figure 3B).

### 3.4. Influence of Mab Anti-GlcCer on Conidia-Peritoneal Macrophages Interaction

**Figure 4:**
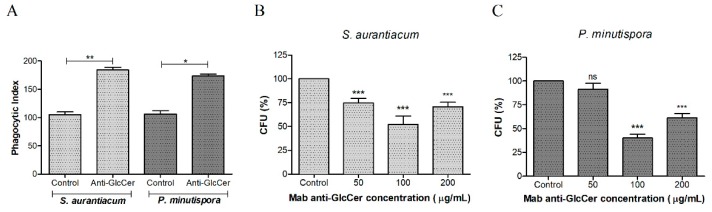
Influence of the anti-GlcCer (Glucosylceramide) Mab (monoclonal antibody) on the phagocytosis of conidia cells by peritoneal macrophages. (**A**) *S. aurantiacum* and *P. minutispora* conidia (10^6^) were incubated with 50, 100, and 200 ug/mL of Mab anti-GlcCer at 37 °C for 1 h. Fungal cells treated with anti-GlcCer MAb were more efficiently internalized, as demonstrated by the phagocytic indices. The phagocytic index values represent the mean ± S.D. of three independent experiments performed in triplicate. (**B,C**) The microbial killing of *S. aurantiacum* (**B**) and *P. minutispora* (**C**) by macrophages was enhanced after treatment with anti-GlcCer Mab. CFU: colony forming units. * *p* < 0.05, ** *p* < 0.03, *** *p* < 0.001.

In order to check whether Mab anti-GlcCer influences host-fungus interaction, phagocytosis and killing assay were performed with conidia (pre-treated or not with Mab) and mice peritoneal macrophages. Mab-treated conidia (100 µg/mL) significantly increased phagocytosis of *S. aurantiacum* and *P. minutispora* compared to the control, suggesting an opsonizing effect (Figure 4A). In addition, the killing assay showed that the opsonized conidia were more efficiently killed by macrophages, at all Mab concentrations for *S. aurantiacum* and mainly at 100 µg/mL of Mab for *P. minutispora*, indicating that anti-GlcCer Mab enhanced the antimicrobial activity of host phagocytic cells (Figure 4B).

### 3.5. Peritoneal Macrophages Activation by GlcCers

**Figure 5:**
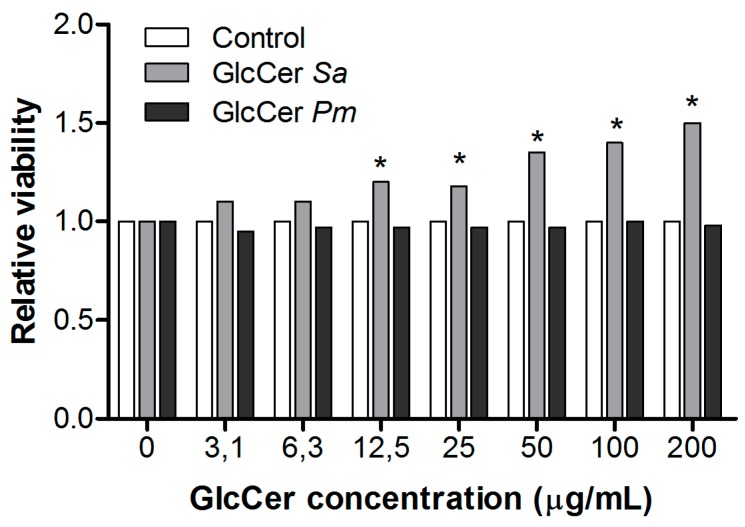
Peritoneal macrophages activation by purified glucosylceramides (GlcCers) from *S. aurantiacum* (Sa) and *P. minutispora* (Pm). Cell viability was measured by neutral-red dye uptake assay, and the results are presented as relative viability compared to the control. * *p* < 0.05.

To analyze whether and how GlcCers from *S. aurantiacum* and *P. minutispora* activate peritoneal macrophages, a neutral-red dye uptake assay was performed to evaluate macrophage metabolism by measuring neutral-red incorporation into lysosomes of viable cells. *S. aurantiacum* GlcCer increased macrophage viability at 12.5, 25, 50, 100, and 200 µg/mL, while *P. minutispora* GlcCer did not alter macrophage viability at any concentration used (Figure 5). These results indicate that GlcCer from both fungi differently stimulates peritoneal macrophages.

**Figure 6:**
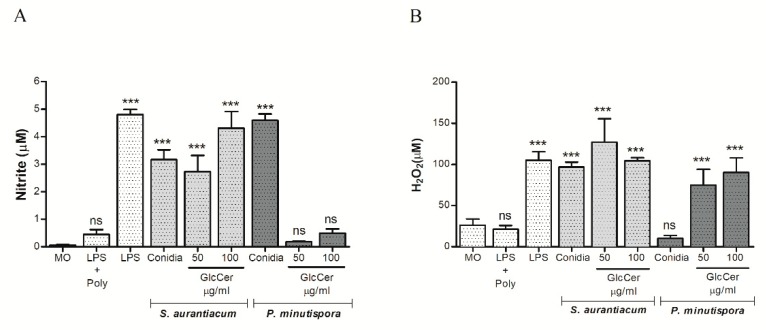
Activation of the oxidative burst in peritoneal macrophages. Macrophages (2.5 × 10^5^) were incubated at 37 °C for 2 h in the presence of *S. aurantiacum* and *P. minutispora* conidia, as well as 50 and 100 mg/mL of purified GlcCer (Glucosylceramide) from both fungi in order to quantify nitrite (**A**) and H_2_O_2_ (**B**). As positive controls, LPS (lipopolysaccharide) (10 ng/well) was used. Data are the mean of duplicate samples from four independent experiments. *p* < 0.001; +Poly, polymyxin added. *** *p* < 0.001.

Macrophage fungicidal activity was also evaluated by nitric oxide (NO) production after incubation with *S. aurantiacum* and *P. minutispora* conidia and purified GlcCer (50 and 100 µg/mL). Conidia of both fungi induced nitric oxide production. Regarding purified GlcCer, only *S. aurantiacum* GlcCer significantly increased nitric oxide production at 50 and 100 µg/mL, while *P. minutispora* GlcCer did not significantly induce NO production, indicating that structural differences could influence NO production (Figure 6A).

Superoxide production was also evaluated, showing that GlcCer from both fungi induced its production at 50 and 100 µg/mL. Interestingly, only *S. aurantiacum* conidia increased superoxide production, while *P. minutispora* conidia did not alter this parameter (Figure 6B). Additionally, *S. aurantiacum* GlcCer induced more superoxide production compared to *P. minutispora* GlcCer (Figure 6B). These results indicate that variations in superoxide production are observed between species.

## 4. Discussion

Glucosylceramide (GlcCer) is an important molecule for fungal growth and virulence. The GlcCer structure is highly conserved among different fungal species. For *Scedosporium/Lomentospora* species, it has already been described in *P. boydii, S. apiospermum* [12,13], and *L. prolificans* [19]. The present work brings the first description of GlcCers in *P. minutispora* and *S. aurantiacum*. The comparison between these two species becomes relevant since *P. minutispora* is a typical environmental isolate with rare cases of human infections, while *S. aurantiacum* is a typical clinical isolate being considered one of the most virulent and resistant *Scedosporium/Lomentospora* species [4,23,24].

The major GlcCer structure found in *P. minutispora* (*m/z* 760) has also been previously found in *Fusarium* species and *A. fumigatus* [25], whereas the one identified in *S. aurantiacum* (*m/z* 734) is similar to *P. boydii* and *S. apiospemum* GlcCers [12,13]. GlcCers isolated from other species belonging to the *Scedosporium/Lomentospora* complex, such as *P. ellipsoidea* and *P. boydii,* presented three molecular species at *m/z* 734, 750, and 762 [14]. These data indicate that GlcCers might vary among species from *Scedosporium/Lomentospora* complex.

Studies performed with *C. neoformans*, *P. boydii*, *S. apiospermum*, *P. angusta,* and *P. ellipsoidea* using monoclonal antibodies against GlcCer described that these sphingolipids are localized on fungal surface, especially as part of the plasma membrane and cell wall [8,12,13,14]. Our data corroborate with the literature, since Mab anti-GlcCer recognized *P. minutispora* and *S. aurantiacum* conidia, as well as on *S. aurantiacum* germ tube. Interestingly, Mab anti-GlcCer is developed using purified *A. fumigatus* GlcCer, therefore, revealing that Mab binds to an epitope conserved among different fungal GlcCers and that differences in Mab recognition could be explained by variation in the exposition of GlcCer on the fungal surface.

It has been shown that GlcCer plays a crucial role in the fungal morphological transition, as also demonstrated for *C. gloeosporioides*, *F. pedrosoi*, *C. albicans*, *A. fumigatus*, *P. boydii,* and *S. apiospermum* [9,10,11,12,13]. Interestingly, the germination of *P. minutispora* and *S. aurantiacum* was not affected by Mab, similarly to what has been described for *Paracoccidioides brasiliensis*, *Histoplasma capsulatum,* and *Sporothrix schenckii* whose fungal growth rates are not affected by anti-GlcCer Mab [26]. These results are different from what is observed for *S. apiospermum*, in which germination is reduced by about 50%, but cell viability is not affected by the Mab [13]. These data suggest that even not affecting directly the morphological transition, Mab binding to GlcCer on the cell surface can influence fungal metabolism.

Monoclonal antibodies anti-GlcCer have been shown to opsonize fungal conidia, as demonstrated in *F. pedrosoi* and *S. apiospermum* models [13,27]. These effects are interesting for fungal clearance since the macrophage cellular response is the most efficient to eliminate fungal infections [28]. Our data corroborate with these studies since Mab anti-GlcCer enhanced macrophages fungicidal activity against *S. aurantiacum* and *P. minutispora*. These data corroborate with the immunofluorescence and the viability assays since the binding of Mab to fungal conidia leads to its opsonization, and the decreased cell viability can contribute to lower fungal survival after macrophage phagocytosis. Bueno and colleagues have recently shown similar results for *P. brasiliensis* using polyclonal anti-glycosphingolipids antibodies, in which opsonization increased macrophage phagocytosis and reduced fungal survival [29].

Since conidia opsonization significantly influences macrophage phagocytosis and killing, we evaluated macrophage activation to check how they eliminate fungal cells. Macrophage action occurs by the release of reactive oxygen and nitrogen species, such as superoxide anions and nitric oxide, leading to fungal digestion inside phagocytic cells [28]. Acidic glycosphingolipids from *P. brasiliensis* also do not increase nitric oxide production [29], suggesting that each fungal species interacts differently with host immune cells depending on the variation of lipid structures. Superoxide has been described as an important substance produced by macrophages during *C. neoformans* clearance. However, *C. neoformans* presents an enzyme, superoxide dismutase, as an evasion mechanism that inactivates superoxide anions and enhances fungal virulence [30]. On the other hand, *H. capsulatum* is killed by bronchoalveolar macrophages through the production of other substances, such as hydrogen peroxide and nitric oxide production [31]. Although ROS production is induced by GlcCer purified from both, *S. aurantiacum* and *P. minutispora*, only conidia of *S. aurantiacum* result in induction. This can be due to the ability of some fungi to block ROS production by macrophages. *Coccidioides* can block nitric oxide production by mice macrophages treated with interferon gamma (IFN-γ) and LPS because of the secretion of an unknown soluble factor [32]. In addition, some fungi, such as *A. fumigatus*, decrease fungicidal effects in macrophages by synthetizing a superoxide dismutase that inhibits the action of superoxide anions [33], a finding that could explain why GlcCer purified from *P. minutispora*, but not its conidia, induces ROS production.

Interesting differences were observed between GlcCer from *S. aurantiacum* and *P. minutispora*, especially regarding the macrophage function experiments. GlcCer from *S. aurantiacum* induced higher macrophage cell viability, intracellular fungal clearance, and the production of fungicidal substances, such as nitric oxide and superoxide, compared to GlcCer from *P. minutispora*. GlcCer structure of *S. aurantiacum* is similar to the one found in *L. prolificans*, which has been also shown to stimulate host immune response [19]. On the other hand, GlcCer of *P. minutispora*, which induced less macrophage effector response, possesses a different ceramide containing a longer fatty acid and an unsaturation (C18:1) when compared to *S. aurantiacum* whose GlcCer is consisted of ceramide with shorter saturated fatty acid (C16:0). It suggests that GlcCer structure influences the stimuli promoted in immune cells. In bacteria, the lipopolysaccharide (LPS) localized in the cell wall is a well-known immune response stimulator. However, differences in LPS structure, especially in the lipid A portion, lead to higher or lower inflammatory responses [34,35]. LPS containing 10-carbon chain in lipid A displays the best cytokine induction, whereas other acyl chain length results in a different cytokine profile [36,37].

Another possible explanation for these differences in fungi-macrophage interaction is the origin of the strains used. *S. aurantiacum*, which induced a higher macrophage activity, is a clinical isolate, while *P. minutispora* is an environmental one. These results corroborate with the study of Caffrey-Carr and colleagues [38], in which the clinical strain of *A. fumigatus* promoted a higher interleukin 1α level in the lungs of infected mice compared to environmental isolates [38]. Our data suggest that the source of isolation (clinical or environmental) could interfere in host-pathogen interaction and, consequently, the immune response profile by the host.

Taken together, the information presented here indicates that GlcCer structure varies among fungal pathogens, and this variation could lead to different biological properties.
